# Tumor Microenvironment Onmyoji: Cytokines with Dual Protumor and Antitumor Roles

**DOI:** 10.34133/cancomm.0008

**Published:** 2026-01-28

**Authors:** Yaxuan Wang, Anqi Lin, Zaoqu Liu, Quan Cheng, Jian Zhang, Peng Luo

**Affiliations:** ^1^ Department of Oncology, Zhujiang Hospital, Southern Medical University, Guangzhou 510282, Guangdong, China.; ^2^ Institute of Basic Medical Sciences, Chinese Academy of Medical Sciences and Peking Union Medical College, Beijing 100730, China.; ^3^ Department of Neurosurgery, Xiangya Hospital, Central South University, Changsha 410008, Hunan, China.; ^4^ National Clinical Research Center for Geriatric Disorders, Xiangya Hospital, Central South University, Hunan, China.; ^5^ School of Clinical Medicine, Li Ka Shing Faculty of Medicine, The University of Hong Kong, Hong Kong, China.

## Abstract

Cytokines are essential components of the tumor microenvironment (TME) and play crucial roles in tumor initiation and progression. As key mediators of interactions between immune cells and tumor cells within the TME, many cytokines exhibit both protumor and antitumor properties. This complex duality, reminiscent of the balance philosophy pursued by “Onmyoji” in traditional Eastern philosophy, which involves observing and regulating opposing forces to achieve harmony, poses marked challenges in translating cytokine therapies from animal studies to clinical applications. More than 20 key cytokines constituting the TME primarily exert their effects through autocrine and paracrine mechanisms: on one hand, they can activate antitumor immune cells, inhibit tumor growth and metastasis, and induce tumor cell apoptosis to exert antitumor effects; on the other hand, they can also recruit abundant immunosuppressive cells, promote angiogenesis, and facilitate the formation of immunosuppressive microenvironments, thereby preventing natural killer and T cells from exerting their cytotoxic antitumor functions. During acute immune responses triggered by tumor antigens, the body typically stimulates dendritic cell maturation and antigen presentation, leading to antitumor immune responses; however, when acute inflammatory reactions are not promptly resolved, they subsequently transform into chronic inflammation, thereby promoting tumor progression and therapeutic resistance, wherein abundant inflammatory cytokines in the TME play crucial roles in this transition. Currently, the major obstacles to cytokine applications in combination immunotherapy are their poor persistence and uncontrolled toxic side effects, resulting in limited therapeutic efficacy; therefore, reducing toxicity while enhancing efficacy has become a top priority in current cytokine therapy-related research. The effectiveness of cytokines exhibits multifactorial regulation influenced by the unique features of the local TME, cytokine concentration, and the responsiveness profiles of target immune effector cells. This review summarizes current research on cytokines with dual protumor and antitumor effects, with a particular focus on the evolution and regulation of their functions during tumor progression, aiming to provide insights for the future development of personalized immunotherapy strategies targeting cytokines.

## Background

Malignant transformation occurs within a sophisticated biological niche where diverse cellular constituents and soluble mediators collectively orchestrate disease progression through interconnected regulatory circuits [1]. This tumor microenvironment (TME) represents a dynamic ecosystem wherein neoplastic cells, infiltrating immune populations, and supporting stromal elements engage in complex crosstalk that ultimately determines therapeutic outcomes [2–5]. Acting as molecular intermediates within these networks, cytokines constitute a diverse family of secreted proteins that facilitate intercellular communication, exhibiting remarkable functional versatility in their capacity to either suppress or promote malignant phenotypes [6]. These molecules exhibit dynamic characteristics including pleiotropy, cascade effects, and transient activity [7–9]. They are capable of exerting marked biological effects at extremely low concentrations, dynamically regulating intercellular interactions within the TME, and playing important roles in multiple processes including tumorigenesis, progression, treatment response, and prognosis [10–12].

Chronic inflammation refers to prolonged inflammatory responses lasting weeks, months, or even years, characterized by sustained production of inflammatory mediators, long-term infiltration of immune cells, and an imbalance between tissue repair and damage [13]. Unlike acute inflammation, which is self-limiting and beneficial, chronic inflammation often loses normal regulatory mechanisms, leading to persistent tissue damage and functional abnormalities [14]. The chronic inflammatory environment is a typical characteristic of the TME, and emerging evidence indicates that persistent inflammatory states constitute key carcinogenic determinants, given that the molecular cascades they initiate can promote the malignant transformation of cells, including DNA lesions, uncontrolled cellular proliferation, apoptosis resistance, and angiogenesis [14,15]. Cytokines, as key signaling proteins in the TME, play a crucial role in regulating immune responses [16]. The pleiotropy of cytokines enables them to play complex roles in the processes of tumor initiation and development. In the field of cancer immunotherapy, researchers are attempting to harness and modulate this characteristic of cytokines to enhance therapeutic efficacy and limit potential side effects [17].

Although researchers are increasingly recognizing the importance of cytokines in the TME, a comprehensive and in-depth understanding of their dual roles in promoting and inhibiting tumor growth remains lacking. Many cytokines possess complex dual regulatory functions in tumor immunity, exerting distinctly different or even opposing effects under varying biological contexts—both promoting antitumor immunity and inducing immunosuppression and tumor progression. This intricate duality, much like the “Onmyoji” concept alluded to in the title of this review—powerful entities capable of exerting opposing influences depending on environmental conditions—reflects the highly context-dependent and plastic nature of cytokine functions. Recent reviews have assessed the dual roles of cytokines in cancer, thereby providing an important foundation for understanding this complex phenomenon [18–21]. However, the current understanding of these cytokine functions remains relatively fragmented, lacking a comprehensive grasp and mechanistic elucidation of their dual roles [22]. Therefore, systematically summarizing and exploring the dual pro- and antitumor functions of tumor-associated cytokines and their regulatory mechanisms is of great importance for deepening our understanding of tumor immune regulation and developing precision immunotherapy strategies.

Cytokines represent an important entry point for tumor immunotherapy [21]. On the one hand, by reshaping the cytokine network within the TME, antitumor immune responses can be enhanced and the efficacy of immunotherapies such as immune checkpoint inhibitors can be improved [23]; on the other hand, targeting specific immunosuppressive cytokines can lead to the development of novel tumor treatment strategies, such as cytokine antibodies and cytokine receptor antagonists [24]. Currently, one of the major obstacles to the application of cytokines in immunotherapy is their toxic side effects, which are dictated by their inherent properties [23]. Through cytokine engineering, researchers can improve their targeting specificity or reduce toxicity, thereby promoting their broader application in immunotherapy [25,26].

This review focuses on the dual regulatory roles of cytokines in the tumor immune microenvironment. First, we provide an overview of the basic composition and general characteristics of cytokines in the TME, with particular emphasis on several representative classes of bifunctional cytokines in tumor immunity, and summarize the research progress regarding their roles in promoting antitumor immunity and inducing immunosuppression. Second, we delve into the molecular mechanisms underlying the dual functions of these cytokines and analyze their cellular and molecular bases of action. Finally, we examine the application prospects of cytokine research in tumor immunotherapy, analyze the challenges faced and potential solutions, and provide insights for the development of next-generation cytokine-based immunotherapeutic regimens. Through an in-depth understanding of the mechanisms of action and regulatory patterns of bifunctional cytokines, we anticipate the development of more precise and effective tumor immunotherapy strategies, which will ultimately provide better therapeutic outcomes for cancer patients.

## Biological Characteristics and Signaling Mechanisms of Cytokines

Cytokines, as a class of small proteins with diverse biological activities, play a central role in intercellular communication and functional regulation within the TME. They not only are abundant in number and diverse in type, but also exhibit complex and varied functions, being capable of precisely regulating the interactions between tumor cells and immune cells. To gain a comprehensive understanding of the mechanisms by which cytokines function in tumor initiation and progression, it is essential to first comprehensively examine the basic biological characteristics of cytokines, their classification systems, and their signal transduction mechanisms within the TME.

### General characteristics of cytokines

Although different cytokines vary in their amino acid sequences and 3-dimensional structures, they share a series of common biological characteristics that determine their functions and regulatory mechanisms in the TME [27].

#### Molecular characteristics and physical properties

First, all cytokines are protein molecules with relatively small molecular weights, typically ranging from 8 to 30 kDa [28]. This small molecular size enables cytokines to freely diffuse through intercellular spaces and body fluids, thereby facilitating their regulatory functions at both short and long distances [29]. Second, cytokines are secreted in soluble forms and can be directly dissolved in extracellular fluid or blood, allowing them to be transported through the circulation to target cell surfaces, and exert their effects without requiring direct cell-to-cell contact [30].

#### Biological activity characteristics

Another important characteristic of cytokines is their high potency, meaning they can produce important biological effects at extremely low concentrations. Most cytokines can bind to high-affinity receptors on target cell surfaces at nanomolar (nmol/l) to picomolar (pmol/l) concentrations, triggering intracellular signal transduction cascades and regulating various biological functions of target cells, including proliferation, differentiation, migration, and the secretion of effector molecules [31]. This high potency enables the organism to precisely regulate the intensity and duration of immune responses and inflammatory reactions with relatively low energy expenditure.

#### Expression of regulation characteristics

Cytokines are prototypical inducible secretory proteins, the synthesis and secretion of which are tightly regulated at both the transcriptional and post-translational levels. Under normal physiological conditions, most cytokines are expressed at low basal levels [32]. Upon exposure to stimuli such as pathogen infection, tissue injury, or stress, the expression of relevant cytokines is rapidly up-regulated, thereby initiating and amplifying the host’s defense responses [33]. Once the stimulatory factors are removed, cytokine expression levels quickly return to baseline, thereby preventing excessive immune responses or inflammatory reactions that could damage the host.

#### Metabolic kinetic characteristics

Cytokines undergo rapid metabolic turnover in vivo and generally have short half-lives, with most being degraded within minutes to hours [28]. This dynamic equilibrium enables the body to precisely regulate cytokine activity in both space and time, allowing immune and inflammatory responses to be rapidly initiated and appropriately terminated.

#### Mode of action characteristics

Although some cytokines can enter the bloodstream via endocrine mechanisms and exert systemic effects, the vast majority primarily function through paracrine or autocrine mechanisms, mediating short-range regulatory effects within the local microenvironment [34].

Whether cytokines exert systemic or local effects is primarily determined by the following factors: First, the molecular weight and structural stability of cytokines are key determinants. Smaller and more stable cytokines (such as certain chemokines) can more readily cross vascular barriers to enter the circulatory system and exert systemic effects, whereas cytokines with larger molecular weights or structural instability tend to act locally near their sites of production [9]. Second, the binding affinity of cytokines for extracellular matrix components markedly influences their diffusion range. Cytokines with strong binding affinity are often sequestered within tissues, forming concentration gradients that guide cell migration and local immune responses [35]. Third, local vascular permeability and lymphatic drainage determine the efficiency with which cytokines enter the systemic circulation. In inflammatory or TMEs, alterations in vascular permeability can markedly influence the range of cytokine action [36]. Finally, the tissue distribution of target cell receptor expression also influences the effective range of cytokine action, as cytokines with restricted receptor expression can only act in specific tissues, even after entering the circulation [37].

This finely regulated mode of action has important biological implications. Local action prevents the systemic dissemination of cytokines, reduces their nonspecific effects on distant tissues and organs, and enhances the specificity and efficacy of immune and inflammatory responses. In contrast, systemic action facilitates the regulation of overall immune status and the coordination of responses between distant tissues. Within the TME, this diversity of action modes provides a crucial foundation for cytokines to exert complex dual regulatory functions and constitutes a key element in understanding their mechanisms of functional switching.

### Major categories of cytokines

Based on these common characteristics of cytokines, researchers have classified cytokines into several major categories according to their primary biochemical features, structural domain characteristics, and functional similarities. Currently, researchers primarily classify cytokines into 6 major categories based on their principal biochemical characteristics: interleukins (ILs), interferons (IFNs), tumor necrosis factors (TNFs), colony-stimulating factors (CSFs), transforming growth factors (TGFs), and chemokines.

#### Interleukins

Early-discovered cytokines were produced by leukocytes and exerted regulatory functions between leukocytes; hence, they were named ILs; however, it is now known that they can also be produced by many other cells including fibroblasts, epithelial cells, and endothelial cells [38,39]. The IL superfamily includes a large number of cytokines, designated as IL-1 to IL-39 [40].

Individual IL species mediate their biological activities through cognate receptor engagement, initiating downstream signaling cascades encompassing multiple transduction networks including Janus kinase (JAK)-signal transducer and activator of transcription (STAT), mitogen-activated protein kinase (MAPK), and nuclear factor kappa B (NF-κB), consequently orchestrating immune cell activation programs, proliferative responses, lineage specification, and effector capabilities within the TME [41–43]. IL functionality within the TME demonstrates context-dependent variability influenced by local molecular concentrations, target cellular populations, and the temporal phase of ongoing immune reactions. These multifactorial determinants constitute the mechanistic basis underlying the bifunctional characteristics observed among numerous IL family members.

#### Interferons

IFNs are primarily produced by monocytes and macrophages, are located on different cell surfaces, and serve as key cytokines for antimicrobial, antitumor, and immune regulatory activities [44,45]. The IFN family primarily consists of 3 types of members: type I IFN (such as IFN-α and IFN-β), type II IFN (such as IFN-γ), and type III IFN (such as IFN-λ), of which type I IFN and type II IFN have been more extensively studied [46–48].

#### Tumor necrosis factor

TNF is a core cytokine in inflammatory responses, primarily expressed by professional antigen-presenting cells (APCs), such as dendritic cells (DCs), B cells, and macrophages, but also expressed by T cells, natural killer (NK) cells, mast cells, and other cell types [49,50]. The TNF and TNF receptor (TNFR) superfamily (TNFSF/TNFRSF) comprises 19 ligands and 29 receptors that participate in regulating cell differentiation, survival, and programmed cell death; however, their most critical function is related to immune regulation, as they are essential for coordinating various mechanisms that drive costimulatory or coinhibitory immune responses. Regulation of TNFSF/TNFRSF may promote antitumor immunity, thus necessitating a better understanding of the fundamental mechanisms of the molecular pathways they mediate in the future [51].

#### Colony-stimulating factors

CSFs are a class of secreted glycoproteins that bind to specific receptors on the surface of hematopoietic stem cells, promoting internal signal transduction and thereby stimulating cell proliferation and differentiation toward specific blood cell lineages [52]. This class of factors primarily includes macrophage-CSF (M-CSF, also known as CSF1), granulocyte-macrophage-CSF (GM-CSF, also known as CSF2), granulocyte-CSF (G-CSF, also known as CSF3), and IL-3 (also known as multi-colony-stimulating factor) [53]. Additionally, erythropoietin and thrombopoietin also belong to the CSF category, due to their promotion of the proliferation and maturation of erythrocyte and platelet precursor cells [52]. CSFs are closely related to tumors, which is specifically manifested as follows: (a) CSFs can promote leukocyte recovery after chemotherapy and reduce infection risk; (b) CSFs can mobilize stem cells into peripheral blood, providing support for hematopoietic stem cell transplantation; (c) CSFs facilitate host immune surveillance against malignancies and augment tumor-directed immunological activities; and (d) CSFs are associated with the pathogenesis of certain myeloid leukemias and may influence disease progression. These effects confer CSFs with important application value in tumor therapy [53].

#### Transforming growth factor

TGF primarily comprises 2 major subtypes: TGF-α and TGF-β. TGF-α belongs to the epidermal growth factor (EGF) family members and shares receptors with EGF, influencing cell proliferation and differentiation [54]. The TGF-β family consists of 3 subtypes: TGFB1, TGFB2, and TGFB3, which play crucial roles in cell proliferation, differentiation, wound healing, embryonic development, and angiogenesis [55]. TGF-β demonstrates exceptional importance across immune homeostasis, stem cell fate determination, and T lymphocyte biology, constituting a central focus within cellular and pathological investigation domains [56]. Understanding the functions of the TGF family is of great importance for developing novel immune combination therapeutic approaches.

#### Chemokines

Chemokines are a class of small molecular cytokines with molecular weights ranging from 8 to 14 kDa that mediate signal transduction through interactions with G protein-coupled receptors on cell surfaces [57]. In this process, chemokines can induce high-affinity adhesion between target cells and endothelial cells, and guide target cell migration toward specific tissue sites according to chemokine concentration gradients [58]. The functions of chemokines can be classified into 2 categories: homeostatic and inflammatory [59]. Homeostatic chemokines are constitutively expressed in specific tissues and participate in regulating leukocyte migration under basal conditions, primarily including CCL14, CCL19, CCL20, CCL21, CCL25, CCL27, CXCL12, and CXCL13 [59]. It should be noted that this classification is not absolute, as CCL20 can also function as a proinflammatory chemokine under specific conditions [60]. Inflammatory chemokines are primarily produced under pathological conditions (such as upon exposure to proinflammatory stimuli including IL-1, TNF-α, lipopolysaccharide, or viruses) and play important roles in inflammatory responses by recruiting immune cells to infiltrate inflammatory sites [59]. Typical inflammatory chemokines include CXCL-8, CCL2, CCL3, CCL4, CCL5, CCL11, and CXCL10. Based on the structure of chemokines and the spacing between 2 cysteine residues at the N-terminus, chemokines can be classified into 4 categories: CXC, CC, C, and CX3C [61].

### Signal regulation of cytokines in tumors

After understanding the basic characteristics and classification of cytokines, it is crucial to gain a comprehensive understanding of the molecular mechanisms by which cytokines exert their functions in the TME. Cytokines serve as important intercellular communication mediators in the TME, which bind to specific receptors on the surface of target cells, thereby triggering intracellular cascade signal transduction, regulating downstream gene expression, and ultimately affecting various biological processes including cell proliferation, differentiation, apoptosis, and effector molecule secretion. Currently, the known signaling pathways activated by cytokine receptors mainly include 3 major categories: JAK-STAT, MAPK, and phosphatidylinositol 3-kinase (PI3K)-protein kinase B (AKT) [62]. In addition, this study also summarizes other signaling pathways that play important roles in cytokine receptor activation.

#### JAK-STAT signaling pathway

Among the principal mechanisms governing cytokine signal transduction, the JAK-STAT cascade represents a paradigmatic pathway linking extracellular stimuli to transcriptional responses [63]. This evolutionarily conserved system demonstrates particular relevance for type I and type II cytokine receptor families, functioning through coordinated interactions between distinct molecular components [64]. The JAK protein family encompasses 4 catalytically active tyrosine kinases (JAK1, JAK2, JAK3, and TYK2) that associate with cytoplasmic receptor domains [65], while the STAT protein cohort consists of 7 transcriptional regulators (STAT1 to STAT4, STAT5A/B, and STAT6) that serve as both signal transducers and deoxyribonucleic acid (DNA)-binding factors [66]. This modular organization enables rapid conversion of membrane-proximal phosphorylation events into nucleus-localized gene expression programs.

When cytokines bind to their corresponding receptors, the JAK proteins associated with the intracellular domain of the receptor are activated and phosphorylate specific tyrosine residues in the intracellular domain of the receptor. These phosphorylation sites can be recognized and bound by the SH2 domain of STATs. Subsequently, JAK further phosphorylates the STAT molecules bound to the receptor, causing them to form homodimers or heterodimers. The activated STAT dimers dissociate from the receptor, rapidly translocate to the nucleus, specifically bind to DNA sequences in the promoter regions of target genes, recruit auxiliary transcription factors and RNA polymerase II, and initiate the transcriptional expression of target genes [66].

#### MAPK signaling pathway

The MAPK signaling pathway represents a class of evolutionarily highly conserved serine/threonine kinase cascade pathways that play crucial roles in complex cellular processes including proliferation, differentiation, development, transformation, and apoptosis [67]. To date, 3 MAPK families have been clearly characterized in mammalian cells: the classical MAPK (also known as ERK), c-Jun N-terminal kinase/stress-activated protein kinase (JNK/SAPK), and p38 kinase [67]. MAP kinases are positioned within protein kinase cascades. Each cascade consists of at least 3 sequentially activated enzymes: MAPK kinase kinase (MAPKKK), MAPK kinase (MAPKK), and MAP kinase (MAPK), with at least 14 MAPKKKs, 7 MAPKKs, and 12 MAPKs having been identified in mammalian cells [68]. Following cytokine receptor activation, the MAPK pathway can be activated through multiple mechanisms. For example, IL-1 receptors and TNFRs can recruit TNF receptor-associated factor family proteins, subsequently activating upstream MAP3K (such as TAK1) of the MAPK pathway, which then sequentially phosphorylates and activates downstream MAP2K and MAPK. Once activated, these terminal kinases modify diverse downstream targets encompassing DNA-binding factors (including AP-1 and NF-κB), cell division regulators (notably p21 and p27), and proapoptotic mediators (such as Bad and Bim), ultimately regulating target gene transcriptional expression and cell fate determination [69]. Indeed, different MAPK branches exert distinct biological functions in specific cytokine signal transduction. For instance, the ERK pathway primarily mediates cell proliferation and survival signals [70], while JNK and p38 MAPK pathways mainly mediate stress responses and proinflammatory/proapoptotic signals [71]. Aberrant MAPK signaling pathways are associated with various inflammatory diseases and tumor development and progression.

#### PI3K-AKT signaling pathway

The PI3K-AKT signaling pathway is one of the most important cellular functional pathways that regulate cell growth, migration, survival, metabolism, and angiogenesis [72], thereby contributing to tumor development and resistance to anticancer therapy [73]. Within this pathway, PI3K is a class of phosphatidylinositol kinases that catalyzes the phosphorylation of PI(4,5)P2 to generate the second messenger PI(3,4,5)P3 [74], while AKT is a class of serine/threonine kinases containing a pleckstrin homology (PH) domain that can specifically bind to PI(3,4,5)P3 and become activated [75]. The intracellular regions of various cytokine receptors (such as IL-2, IL-3, and IL-4 receptors) contain specific tyrosine phosphorylation sites that can be recognized and bound by the SH2 domain of the regulatory subunit of intracellular PI3K [76–78]. Upon receptor activation, the catalytic subunit phosphorylates PI(4,5)P2 to generate PI(3,4,5)P3, which subsequently recruits AKT to the vicinity of the plasma membrane, where it is phosphorylated and activated by protein kinases such as phosphoinositide-dependent kinase 1 (PDK1) [79]. Subsequently, activated AKT can further phosphorylate and regulate the activity of various substrate proteins including mTOR, FOXO, GSK3β, and Bad, promoting cell proliferation, survival, and metabolism while inhibiting apoptosis [80–82]. The PI3K-AKT signaling pathway plays a crucial role in cytokine-induced cell proliferation and survival, metabolic reprogramming, and immune regulation. Aberrant activation of this pathway can lead to the occurrence and development of various metabolic diseases, autoimmune diseases, and tumors [83]. In fact, PI3K and AKT have now become important targets for tumor therapeutic intervention.

#### Other signaling pathways

Additionally, the following signaling pathways also play important roles in the TME: the NF-κB pathway is frequently activated by proinflammatory cytokines such as TNF-α and IL-1β, playing a crucial role in inflammatory responses and immune regulation [84,85]; the TGF-β/Smad pathway exhibits dual roles in tumor progression, affecting tumor growth and immune suppression [86,87]. The Wnt/β-catenin and Notch pathways participate in regulating tumor stem cell characteristics, angiogenesis, and immune cell function [88,89]. Furthermore, the Hippo/YAP pathway also plays an important role in tumor cell proliferation and TME stromal remodeling [90]. These pathways are intricately interwoven, collectively shaping the complex TME and influencing tumor progression, metastasis, and therapeutic responses. An in-depth understanding of the mechanisms and interrelationships of these pathways will facilitate the development of novel cancer therapeutic strategies and improve patient outcomes.

In summary, cytokines, as key regulatory molecules in the TME, possess complex biological properties and diverse classifications. They exert their functions through multiple signaling pathways, forming an intricate regulatory network. These pathways are intricately interwoven, collectively shaping the complex TME and influencing tumor progression, metastasis, and therapeutic responses. An in-depth understanding of the mechanisms and interrelationships of these pathways will facilitate the development of novel cancer therapeutic strategies and improve patient outcomes.

Based on our understanding of the fundamental characteristics and regulatory mechanisms of cytokines, we will specifically explore the functional performance of different cytokines in the TME in the following chapters, including their antitumor effects, protumor effects, and complex dual regulatory functions.

## Cytokines with Unidirectional Antitumor or Protumor Functions

### Cytokines with antitumor effects

**Fig. 1.**
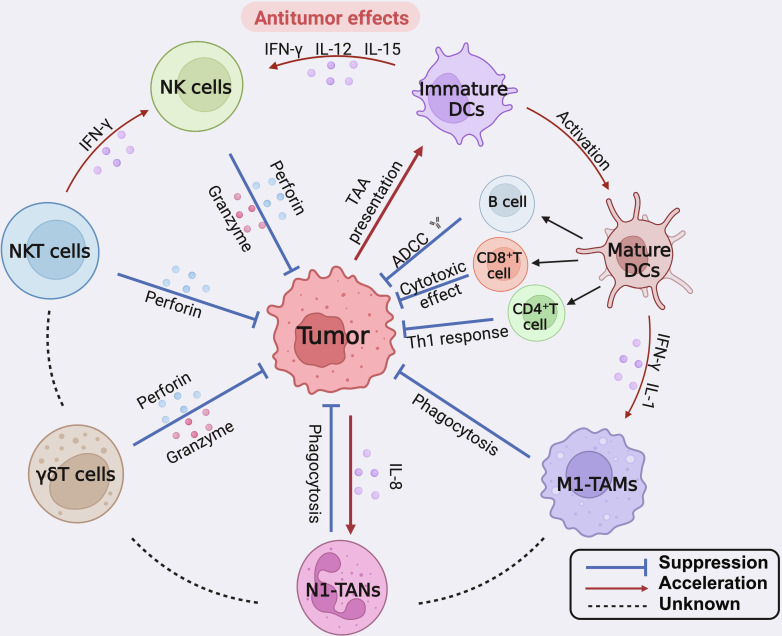
The antitumor mechanism of cytokines. Cytokines contribute to the antitumor response through several key pathways and interactions with different immune cells. These include the following: (a) Natural killer (NK) cells: Cytokines such as IFN-γ, IL-12, and IL-15 activate NK cells. Activated NK cells then release perforin and granzyme, which induce apoptosis in tumor cells. (b) Natural killer T (NKT) cells: These cells also release perforin in response to cytokines, contributing to the direct killing of tumor cells. (c) γδ T cells: Similar to NK and NKT cells, γδ T cells release perforin and granzyme to target and kill tumor cells. (d) Immature dendritic cells: Cytokines activate immature dendritic cells, which then mature and present tumor-associated antigens (TAAs) to other immune cells, enhancing the adaptive immune response. (e) Mature dendritic cells: These cells facilitate the activation of B cells, CD8^+^ T cells, and CD4^+^ T cells through antigen presentation. This activation leads to antibody-dependent cellular cytotoxicity (ADCC), cytotoxic effects, and a Th1 response, all of which contribute to the antitumor immune response. (f) M1 tumor-associated macrophages (M1-TAMs): Cytokines like IFN-γ and IL-1 activate M1-TAMs, which then engage in phagocytosis of tumor cells and secrete factors that promote inflammation and further immune activation. (g) Neutrophils (N1-TANs): These cells, activated by IL-8 and other cytokines, also participate in phagocytosis and contribute to the destruction of tumor cells. Overall, cytokines orchestrate a multifaceted immune response involving various cell types, enhancing both the innate and adaptive immune systems’ ability to target and eliminate tumor cells. Abbreviations: ADCC, antibody-dependent cellular cytotoxicity; CD4^+^ T cell, CD4-positive T cell; CD8^+^ T cell, CD8-positive T cell; DCs, dendritic cells; IFN-γ, interferon gamma; IL-1, interleukin-1; IL-8, interleukin-8; IL-12, interleukin-12; IL-15, interleukin-15; M1-TAMs, M1 tumor-associated macrophages; N1-TANs, N1 tumor-associated neutrophils; NK cells, natural killer cells; NKT cells, natural killer T cells; TAAs, tumor-associated antigens; Th1, T helper 1; γδ T cells, gamma delta T cells.

Multiple cytokines with distinct antitumor activities have been identified; these cytokines regulate the body’s antitumor immunity through various mechanisms, inhibit tumor growth and metastasis, and serve as important candidate drugs for tumor combination immunotherapy strategies (Fig. 1 and Table 1).

**Table 1.** Summary of major cytokines and their roles in the tumor microenvironment

Cytokine	Antitumor mechanisms	Protumor mechanisms
Predominantly antitumor		
IL-12	Promotes Th1 differentiation, activates NK cells and CTLs	None
IL-18	Stimulates IFN-γ production, inhibits tumor metastasis	None
IL-21	Enhances memory T cell function	None
IL-24	Selectively induces tumor cell apoptosis	None
IFN-α	Up-regulates MHC-I, induces cell cycle arrest	None
IFN-β	Inhibits proliferation, enhances NK/CTL activity	None
TRAIL	Selectively induces tumor cell apoptosis	None
FasL	Fas/FasL pathway-mediated apoptosis	None
Predominantly protumor		
IL-4	None	Induces M2 polarization, suppresses Th1 responses
IL-35	None	Induces T cell exhaustion, immunosuppression
IL-8	None	Promotes angiogenesis, recruits neutrophils
CXCL12	None	Promotes metastasis, activates survival signals
CCL2	None	Recruits TAMs, promotes metastasis
TGF-α	None	Activates EGFR, promotes proliferation
CSF1	None	Promotes M2 macrophage polarization, immunosuppression
VEGF	None	Promotes angiogenesis, immunosuppression
Dual functions		
IL-1β	Promotes DC maturation, activates T cells	Chronic inflammation, recruits immunosuppressive cells
IL-2	Promotes effector T cell proliferation	Promotes Treg expansion, induces T cell exhaustion
IL-6	Promotes acute inflammation, activates NK/CTLs	Induces EMT, maintains stem cell properties
IL-7	Promotes T/B cell development	Promotes tumor cell survival
IL-10	Enhances CAR-T function, promotes CD8^+^ T cells	Immunosuppression, promotes tumor metastasis
IL-15	Activates NK cells, maintains memory T cells	Establishes autocrine loops (hematologic malignancies)
IL-17	Recruits DCs, promotes antitumor immunity	Induces EMT, promotes angiogenesis
IL-22	Maintains epithelial barrier	Promotes metastasis, impairs NK function
IL-27	Inhibits angiogenesis, induces apoptosis	Promotes tumor survival (context-dependent)
IFN-γ	Direct cytotoxicity, enhances immunogenicity	Induces PD-L1, promotes immune escape
TGF-β	Inhibits proliferation, induces apoptosis (early)	Promotes EMT, immunosuppression (late)
GM-CSF	Promotes DC differentiation, enhances immunity	Activates immunosuppressive cells
TNF-α	Vascular disruption, direct cytotoxicity	Chronic inflammation, promotes metastasis

CAR-T, chimeric antigen receptor T cells; CCL2, C-C motif chemokine ligand 2; CSF1, colony-stimulating factor 1; CTLs, cytotoxic T lymphocytes; CXCL12, C-X-C motif chemokine ligand 12; DCs, dendritic cells; EGFR, epidermal growth factor receptor; EMT, epithelial–mesenchymal transition; FasL, Fas ligand; GM-CSF, granulocyte-macrophage colony-stimulating factor; IFN, interferon; IL, interleukin; MHC-I, major histocompatibility complex class I; NK, natural killer; PD-L1, programmed death-ligand 1; TAMs, tumor-associated macrophages; TGF, transforming growth factor; TNF, tumor necrosis factor; TRAIL, TNF-related apoptosis-inducing ligand; VEGF, vascular endothelial growth factor

#### IL family

IL-12 is a heterodimeric proinflammatory cytokine that induces IFN-γ production, facilitates Th1 cell differentiation, and establishes a connection between innate and adaptive immunity, thereby enhancing cellular immunity [91]. IL-12 is primarily secreted by monocytes, macrophages, and DCs, while some tumor cells can also produce IL-12 [92]. Its antitumor mechanisms include activating NK cells and cytotoxic T lymphocytes (CTLs), promoting the production of antitumor cytokines such as IFN-γ, establishing effective Th1-type immune responses, and enhancing tumor-specific T cell activation by promoting DC maturation and enhancing antigen presentation capacity [93,94].

IL-18 stimulates NK cells and T cells to produce IFN-γ, thereby inhibiting tumor cell adhesion and metastasis [95]. IL-18 is primarily secreted by activated macrophages, DCs, and some tumor cells [96]. Its mechanism of action involves binding to the IL-18 receptor, activating the MyD88–NF-κB signaling pathway, promoting NK cell and T cell activation, enhancing cytotoxic functions, and inducing the production of various antitumor cytokines [97].

IL-21 induces early differentiation phenotypes in memory T cells and synergizes with IL-7 or IL-15 to promote their proliferation and survival, resulting in tumor-specific T cells with enhanced in vivo antitumor efficacy under its induction; therefore, IL-21-mediated ex vivo programming and activation of T cells hold promise for improving the therapeutic efficacy of adoptive T cell transfer therapy [98]. IL-21 is primarily produced by activated CD4^+^ T cells and NKT cells, and regulates T cell and B cell differentiation through activation of the JAK1/3-STAT3 signaling pathway, promotes the generation of CTLs, and enhances NK cell cytotoxic activity [99].

IL-24 selectively induces tumor cell apoptosis and inhibits tumor growth and angiogenesis [100]. IL-24 can be produced by various cell types, including activated immune cells, fibroblasts, and some normal epithelial cells [101]. Its unique feature lies in its selective toxicity toward tumor cells, specifically inducing malignant cell death through the activation of endoplasmic reticulum (ER) stress responses and mitochondrial apoptotic pathways, while being virtually harmless to normal cells [101]. Additionally, IL-24 can inhibit tumor angiogenesis and block the nutritional supply to tumors [102].

#### IFN family

IFN-α up-regulates major histocompatibility complex (MHC) class I molecule expression on tumor cells, enhances tumor immunogenicity, and induces tumor cell cycle arrest and apoptosis [103]. IFN-α is primarily produced in large quantities by plasmacytoid DCs following viral infection or Toll-like receptor (TLR) activation, and can also be secreted by monocytes and macrophages [104]. As a pleiotropic antitumor cytokine, IFN-α plays an important role in the treatment of various tumor types, including hematologic malignancies (leukemia and lymphoma), melanoma, renal cell carcinoma, and Kaposi’s sarcoma, by activating the JAK-STAT signaling pathway to regulate tumor cell death, inhibit tumor angiogenesis, and enhance antitumor immunity [105]. Its mechanisms include direct antiproliferative and proapoptotic effects, as well as indirect antitumor effects through enhancing antigen presentation and activating immune cells [106].

IFN-β inhibits tumor cell proliferation, induces tumor cell apoptosis, and enhances the cytotoxic activity of NK cells and CTLs [107]. IFN-β is primarily produced by fibroblasts, endothelial cells, and certain immune cells, particularly upon viral infection or inflammatory stimulation [108]. IFN-β activates the IFNAR1/2 receptor complex and downstream JAK-STAT signaling pathway, induces the expression of antiviral and antitumor genes, directly inhibits tumor cell proliferation, simultaneously enhancing the cytotoxic functions of NK cells and CTLs, and inhibits tumor angiogenesis [109].

#### TNF family

TNF-related apoptosis-inducing ligand (TRAIL) selectively induces tumor cell apoptosis with minimal toxicity to normal cells [110]. TRAIL can be produced by various immune cells, including NK cells, CTLs, macrophages, and DCs, and can also be expressed by some normal tissue cells [111]. TRAIL specifically activates the extrinsic apoptotic signaling pathway by binding to death receptors (DR4/DR5) on the tumor cell surface, recruiting adaptor proteins such as Fas-associated protein with death domain (FADD), initiating the caspase-8/10 cascade response, and ultimately leading to the programmed death of tumor cells [112]. Since normal cells typically express fewer death receptors or possess stronger apoptosis resistance mechanisms, TRAIL exhibits relatively low toxicity to normal cells [112].

Fas ligand (FasL) can not only specifically induce tumor cell apoptosis through the Fas/FasL pathway, but also mediate nonspecific “bystander” killing effects on antigen-negative tumor cells. This local bystander cytotoxicity holds promise for overcoming tumor heterogeneity and improving tumor immunotherapy efficacy [113]. FasL is primarily expressed by activated CTLs and NK cells, and induces cell apoptosis by binding to Fas receptors on the surface of target cells and activating death signal transduction [114,115]. The mechanism of FasL-mediated “bystander effect” involves danger signal molecules released by apoptotic cells, which can activate surrounding immune cells and expand the killing range [116].

The common characteristic of these antitumor cytokines is that they generate antitumor immune responses by activating acute inflammatory reactions and stimulating the maturation and antigen presentation of DCs [117]. When DCs bind to TLR agonists or recognize specific tumor-associated antigens, they can be induced to activate and mature [118]. Mature DCs play an indispensable role in the process of immune system recognition and clearance of tumor cells by capturing and presenting tumor antigens, activating tumor antigen-specific T lymphocytes, and secreting proinflammatory and antitumor cytokines [119]. Furthermore, these cytokines can also promote M1 polarization of tumor-associated macrophages (TAMs), recruit and activate NK cells, thereby forming an effective antitumor immune network [120–123].

### Protumor cytokines

**Fig. 2.**
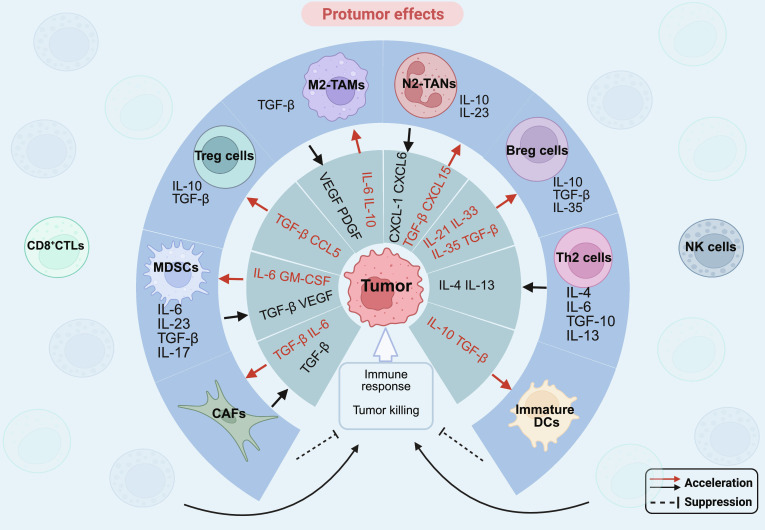
The protumor mechanism of cytokines. Cytokines contribute to protumor effects through several pathways and interactions with various immune and stromal cells, which can lead to immune suppression and tumor progression. These mechanisms include the following: (a) M2-TAMs release TGF-β, a cytokine that promotes immune suppression and aids in tumor progression. (b) N2-TANs secrete IL-10 and IL-23, which inhibit effective immune responses and support tumor growth. (c) Regulatory T (Treg) cells produce IL-10 and TGF-β, both of which suppress the activity of cytotoxic T cells and other immune cells that would otherwise attack the tumor. (d) Regulatory B (Breg) cells release IL-10, TGF-β, and IL-35, which suppress antitumor immunity. (e) Th2 cells release IL-4, IL-6, TGF-β, and IL-13, cytokines that promote a protumor environment by inhibiting the Th1 response and fostering tumor cell survival. (f) Myeloid-derived suppressor cells (MDSCs) release cytokines like IL-6, IL-23, TGF-β, and IL-17, which suppress cytotoxic T cells and NK cells, allowing tumors to evade immune destruction. (g) Cancer-associated fibroblasts (CAFs) produce TGF-β and other factors like IL-6, creating a supportive stroma for tumor growth and further suppressing immune responses. Overall, these cytokines and cell interactions create an environment that promotes tumor growth, inhibits effective immune responses, and supports tumor survival and metastasis. Abbreviations: Breg cells, regulatory B cells; CAFs, cancer-associated fibroblasts; CCL5, C-C motif chemokine ligand 5; CD8^+^CTLs, CD8+ cytotoxic T lymphocytes; CXCL-1, C-X-C motif chemokine ligand 1; CXCL6, C-X-C motif chemokine ligand 6; CXCL15, C-X-C motif chemokine ligand 15; DCs, dendritic cells; GM-CSF, granulocyte-macrophage colony-stimulating factor; IL-4, interleukin-4; IL-6, interleukin-6; IL-10, interleukin-10; IL-13, interleukin-13; IL-17, interleukin-17; IL-21, interleukin-21; IL-23, interleukin-23; IL-33, interleukin-33; IL-35, interleukin-35; M2-TAMs, M2 tumor-associated macrophages; MDSCs, myeloid-derived suppressor cells; N2-TANs, N2 tumor-associated neutrophils; NK cells, natural killer cells; PDGF, platelet-derived growth factor; TGF-β, transforming growth factor beta; Th1, T helper 1; Th2 cells, T helper 2 cells; Treg cells, regulatory T cells; VEGF, vascular endothelial growth factor.

Immunosuppressive cytokines predominate in the TME, which not only suppress the host’s antitumor immune responses but also promote tumor cell survival and proliferation, ultimately leading to malignant tumor progression. In addition to tumor cells that secrete immunosuppressive factors, various heterogeneous immune cells infiltrating the TME also serve as important sources of these factors (Fig. 2).

#### IL family

IL-4 exerts definite protumor effects in multiple tumor types. IL-4 is primarily secreted by Th2 cells, mast cells, eosinophils, and macrophages, while some tumor cells can also produce IL-4 [124]. Studies have demonstrated that in mouse models of colon and breast cancer, blocking IL-4 can reduce the generation of immunosuppressive M2 phenotypes and myeloid-derived suppressor cells (MDSCs) in the TMEs and improve tumor-specific CD8^+^ T cell responses [125]. IL-4 promotes tumor cell survival and proliferation by activating the STAT6 signaling pathway; the underlying mechanisms involve the activation of key pathways such as AKT and NF-κB, a phenomenon particularly prominent in prostate cancer cells [126]. Furthermore, IL-4 can also trigger the MAPK pathway and induce proliferation of various tumor cells, including head and neck squamous cell carcinoma, breast cancer, and ovarian cancer, through JNK activation [127]. The protumor mechanisms of IL-4 also include inducing macrophage polarization toward the M2 phenotype, suppressing Th1-type immune responses, and promoting angiogenesis [128].

IL-35 is a newly discovered inhibitory cytokine primarily secreted by regulatory T cells (Tregs) and regulatory B cells (Bregs), while some tumor cells can also produce IL-35 [129,130]. Current evidence indicates that tumor-derived IL-35 extensively participates in protumor activities across different cellular environments, contributing to the formation of immunosuppressive TMEs by inhibiting tumor-infiltrating lymphocyte infiltration and effector cell proliferation [131]. IL-35 secreted by Tregs in the TME accelerates intratumoral T cell exhaustion by up-regulating the expression of several inhibitory receptors and exhaustion-related genes in tumor-infiltrating CD8^+^ T cells, thereby suppressing effective antitumor immunity [132]. Studies have shown that IL-35 expression is highly elevated in both mouse and human pancreatic tumor samples and specifically inhibits CD8^+^ T cell activity through STAT3-dependent mechanisms [132]. Overexpression of IL-35 is also associated with human gastric cancer progression, and it participates in gastric cancer cell proliferation by up-regulating Ki67 expression and reducing cell apoptosis [133].

#### TGF family

TGF-α belongs to the EGF family members and shares receptors with EGF, thereby affecting cell proliferation and differentiation [54]. TGF-α is primarily produced by various epithelial cells, fibroblasts, and tumor cells [134]. TGF-α stimulates tumor cell proliferation, survival, and migration by activating the epidermal growth factor receptor (EGFR) signaling pathway, which subsequently promotes downstream activation of the RAS-MAPK and PI3K-AKT pathways [134]. TGF-α can also promote angiogenesis and extracellular matrix remodeling, thereby creating favorable conditions for tumor invasion and metastasis [135].

#### Chemokine family

IL-8 is an important neutrophil chemokine that can be produced by various cells, including tumor cells, macrophages, endothelial cells, and fibroblasts [136]. Through binding to CXCR1 and CXCR2 receptors, IL-8 activates downstream PI3K-AKT and MAPK signaling pathways [137], not only recruiting neutrophils to infiltrate the TME but also directly promoting tumor cell proliferation, invasion, and angiogenesis [138]. IL-8 can also induce cancer stem cell (CSC)-like properties, promoting malignant tumor progression and therapeutic resistance [139].

CXCL12 is primarily secreted by bone marrow stromal cells, fibroblasts, and endothelial cells, with some tumor cells also capable of production [140]. Through binding to the CXCR4 receptor, CXCL12 activates multiple signaling pathways, including the PI3K-AKT, MAPK, and JAK-STAT pathways, thereby promoting tumor cell survival, proliferation, and metastasis [141]. The CXCL12/CXCR4 axis plays a crucial role in tumor metastasis, particularly in organs with high CXCL12 expression such as bone marrow, lungs, and liver [142].

CCL2 is primarily secreted by monocytes, macrophages, endothelial cells, and tumor cells [143]. Through binding to the CCR2 receptor, CCL2 recruits monocytes to migrate to tumor sites and promotes their differentiation into TAMs [143]. These TAMs primarily exhibit M2-type polarization and secrete various protumor factors, including IL-10, TGF-β, and vascular endothelial growth factor (VEGF), thereby creating an immunosuppressive microenvironment that promotes tumor growth and metastasis [144].

#### CSF and VEGF

CSF1 is primarily secreted by macrophages, fibroblasts, and tumor cells. CSF1 promotes monocyte differentiation into macrophages and regulates macrophage polarization states by binding to the CSF1 receptor [145]. In the TME, CSF1 primarily promotes the formation and maintenance of M2-type macrophages, which possess immunosuppressive functions and can inhibit T cell activity and promote angiogenesis and tissue remodeling, thereby supporting tumor growth and metastasis [146].

VEGF is a key proangiogenic factor primarily secreted by tumor cells, macrophages, fibroblasts, and endothelial cells. VEGF stimulates endothelial cell proliferation, migration, and blood vessel formation by binding to VEGFR receptors and activating downstream PI3K-AKT, MAPK, and PLC-γ signaling pathways, thus providing nutritional support for tumor growth [147]. VEGF can also increase vascular permeability, facilitating tumor cell entry into the circulatory system and creating conditions for metastasis. Additionally, VEGF possesses immunosuppressive properties and can inhibit DC maturation and T cell function.

The common characteristic of these protumor cytokines is the induction of chronic inflammation, which is their primary mechanism for promoting tumor progression. There is a clear causal relationship between chronic inflammation and tumor development and progression [148]. Chronic inflammatory responses that are persistent, lack precise regulation, and cannot be completely eliminated can promote malignant progression of precancerous lesions through multiple mechanisms, accelerating the progression from carcinoma in situ to invasive and metastatic cancer [149–151]. Once an inflammatory TME is established, inflammatory factors derived from tumor cells or stromal cells can promote tumor cell proliferation and extend their survival time by activating oncogenes and suppressing tumor suppressor gene expression [152]. In this microenvironment, tumor cells can also recruit numerous immunosuppressive cells (such as MDSCs, Tregs, Bregs, and M2 TAMs, etc.) by secreting various inflammatory cytokines; these immunosuppressive cells, in turn, provide a rich proangiogenic and protumor microenvironment while preventing innate immunity and T cell antitumor immunity [117,153].

The complex cytokine network in the TME plays a crucial role in tumor immune regulation and tumor progression. Antitumor cytokines primarily function through mechanisms such as activating acute inflammatory responses, enhancing immune cell function, and directly killing tumor cells, while protumor cytokines promote tumor progression through pathways including establishing chronic inflammation, forming immunosuppressive microenvironments, and supporting angiogenesis. A comprehensive understanding of the mechanisms of action of different cytokines in the TME will facilitate the development of novel tumor immunotherapy strategies targeting cytokines. In the following sections, we will focus on discussing bifunctional cytokines that can exert opposite effects under different conditions; the complexity of these cytokines presents new challenges and opportunities for tumor immunotherapy.

## Cytokines with Dual Antitumor and Protumor Effects

**Fig. 3.**
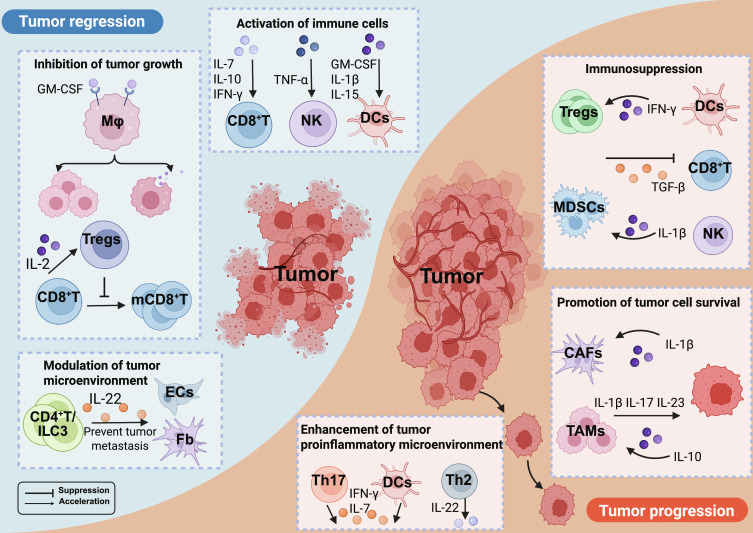
Cytokines with dual roles in tumor immune microenvironment (TIME). Tumor regression: (a) Inhibition of tumor growth: Macrophages (MΦ): Activated by GM-CSF, macrophages inhibit tumor growth by killing tumor cells. CD8^+^ T cells and Tregs: CD8^+^ T cells secrete IL-2, which activates Tregs. Tregs then suppress the differentiation of CD8^+^ T cells into memory CD8^+^ T cells (mCD8^+^T), limiting the antitumor immune response. (b) Activation of immune cells: CD8^+^ T cells: Cytokines like IL-7, IL-10, and IFN-γ promote the activation and proliferation of CD8^+^ T cells to target tumor cells. NK cells: TNF-α enhances the activation of NK cells to directly kill tumor cells. Dendritic cells (DCs): Cytokines like GM-CSF, IL-1β, and IL-15 activate dendritic cells to promote antigen presentation and enhance immune responses. (c) Modulation of the tumor microenvironment: ILC3 and CD4^+^ T cells: Secrete IL-22, which modulates endothelial cells (ECs) and fibroblasts (Fb) to prevent tumor metastasis. Tumor progression: (a) Immunosuppression: DCs: Accelerate Tregs by releasing IFN-γ, and Tregs inhibit CD8^+^ T cells through TGF-β. NK cells and MDSCs: NK cells secrete IL-1β, which acts on MDSCs. In turn, MDSCs secrete TGF-β, which suppresses the function of CD8^+^ T cells, leading to immune evasion by the tumor. (b) Promotion of tumor cell survival: CAFs: IL-1β secreted by other cells in the tumor microenvironment activates CAFs. CAFs then contribute to tumor cell survival by promoting tumor-supportive conditions. TAMs: Similarly, IL-10 secreted by other cells acts on TAMs. TAMs subsequently secrete IL-1β, IL-17, and IL-23, which enhance tumor progression by suppressing antitumor responses. (c) Enhancement of the proinflammatory tumor microenvironment: DCs, Th17, and Th2 cells: DCs, along with Th17 and Th2 cells, contribute to a proinflammatory environment through the secretion of cytokines like IL-7, IFN-γ, and IL-22. This supports tumor growth by creating conditions that promote chronic inflammation and immune modulation. In summary, cytokines have complex roles in both promoting tumor regression by enhancing immune responses and contributing to tumor progression by creating an immunosuppressive microenvironment and supporting tumor cell survival and growth. Abbreviations: CAFs, cancer-associated fibroblasts; CD4^+^ T cells, CD4-positive T cells; CD8^+^ T cells, CD8-positive T cells; DCs, dendritic cells; ECs, endothelial cells; Fb, fibroblasts; GM-CSF, granulocyte-macrophage colony-stimulating factor; IFN-γ, interferon gamma; IL-1β, interleukin-1 beta; IL-2, interleukin-2; IL-7, interleukin-7; IL-10, interleukin-10; IL-15, interleukin-15; IL-17, interleukin-17; IL-22, interleukin-22; IL-23, interleukin-23; ILC3, innate lymphoid cells type 3; mCD8^+^T, memory CD8^+^ T cells; MDSCs, myeloid-derived suppressor cells; MΦ, macrophages; NK cells, natural killer cells; TAMs, tumor-associated macrophages; TGF-β, transforming growth factor beta; Th2, T helper 2; Th17, T helper 17; TIME, tumor immune microenvironment; TNF-α, tumor necrosis factor alpha; Tregs, regulatory T cells.

In the preceding sections, we have individually discussed cytokines with distinct antitumor or protumor effects. However, an important finding in tumor immunology research is that a considerable number of cytokines do not solely exert promotive or inhibitory effects during tumor initiation and progression, but rather exhibit complex dual functional characteristics. These cytokines can both inhibit tumor growth and promote tumor progression under specific conditions, with their functional manifestations being highly dependent on multiple factors, including the developmental stage of the tumor, cytokine concentration, the state of the TME, and the target cell types they act upon and the specific receptors they activate (Fig. 3).

This functional duality poses substantial challenges for understanding the relationship between cytokines and tumors, while simultaneously providing new insights and opportunities for developing cytokine-based personalized tumor immunotherapy. An in-depth understanding of the mechanisms of action and regulatory patterns of these dual-function cytokines is of great importance for accurately predicting therapeutic outcomes, optimizing treatment timing and dosage, and reducing adverse reactions. In the following sections, we will systematically elucidate these key cytokines with dual effects based on cytokine family classifications.

### IL family

#### IL-1β

IL-1β serves as a core regulatory factor in inflammatory responses and exhibits marked dual regulatory roles in tumor immunity. In acute inflammation and early immune responses, IL-1β primarily exerts antitumor functions; however, under chronic inflammatory conditions, it tends to promote tumor progression [21].

Antitumor mechanisms: IL-1β orchestrates multifaceted antimalignancy programs through several interconnected pathways. Initially, this inflammatory mediator facilitates the functional maturation of immature dendritic cells (iDCs), augmenting their capacity for efficient antigen processing and presentation, consequently priming robust tumor-specific adaptive responses [154]. Additionally, IL-1β serves as an indispensable cofactor in memory T cell development, operating through IL-1 receptor (IL-1R)-MyD88 signaling cascades that program both CD4^+^ and CD8^+^ populations for sustained immunological memory [155]. These long-lived memory populations constitute a critical component of durable antitumor surveillance, providing rapid recall responses upon subsequent malignant cell encounters [156]. Furthermore, IL-1β induces the differentiation and expansion of effector T cells, enhances T cell-mediated adaptive antitumor immune responses, and maintains T cell survival and function [117].

Protumor mechanisms: However, in the context of chronic inflammation, sustained high levels of IL-1β expression exhibit pronounced protumor effects. IL-1β drives chronic persistent inflammatory responses and promotes tumorigenesis and development by continuously activating NF-κB and MAPK signaling pathways [157–159]. Specific mechanisms include (a) activating endothelial cells and increasing vascular permeability [160]; (b) stimulating tumor angiogenesis, with increased VEGF secretion by tumor cells and markedly enhanced vascular density [161–163]; (c) recruiting immunosuppressive cells—in head and neck squamous cell carcinoma models, inflammasome activation leads to up-regulated IL-1β expression, thereby forming immunosuppressive networks by inducing MDSCs, TAMs, and Tregs [164]; and (d) promoting tumor metastasis by up-regulating vascular cell adhesion molecule-1 expression on hepatic sinusoidal endothelium, thus facilitating tumor cell adhesion and metastasis [165].

Dynamic changes during disease progression: The expression levels and functions of IL-1β exhibit distinct biphasic patterns of change during tumor progression. In early-stage tumors, IL-1β is primarily and transiently secreted by tumor-infiltrating macrophages and DCs, predominantly promoting acute inflammatory responses and antitumor immunity [166]. As tumors progress to intermediate and advanced stages, continuous tissue damage and necrosis lead to sustained elevation of IL-1β levels; at this juncture, it primarily exerts protumor effects. The key to this transition lies in the shift in inflammatory nature from acute to chronic, as well as overall changes in the state of the immune microenvironment [167].

#### IL-2

IL-2 has traditionally been recognized as a T cell growth factor that plays a crucial role in antitumor immunity; however, recent studies have revealed that it also possesses complex dual regulatory functions [168].

Antitumor mechanisms: The antitumor effects of IL-2 are primarily manifested through promoting the proliferation, differentiation, and survival of effector T cells [169]. During the early stages of tumor immune response, when IL-2 concentrations are relatively low, IL-2 primarily promotes the expansion and activation of antitumor effector T cells, enhancing their capacity to kill tumor cells. IL-2 activates the JAK1/3-STAT5 signaling pathway, up-regulates the expression of antiapoptotic protein Bcl-2, prolongs T cell survival, and promotes T cell proliferation [170]. Moreover, IL-2 can enhance the cytotoxic functions of NK cells and CTLs, thereby improving the body’s capacity for tumor surveillance and clearance [171].

Protumor mechanisms: However, the protumor effects of IL-2 are primarily mediated through the following mechanisms: First, IL-2 is crucial for the growth, development, and functional maintenance of Tregs; high concentrations of IL-2 can promote Treg expansion and activation, thereby suppressing antitumor immune responses [172]. Second, during the intermediate and late stages of tumor development, IL-2 secreted by CD4^+^ T cells in the TME can activate the STAT5 signaling pathway in CD8^+^ T cells, inducing up-regulation of tryptophan hydroxylase 1 (TPH1) expression, which ultimately leads to increased expression of immune checkpoint molecules such as PD-1, TIM-3, and LAG-3, resulting in T cell exhaustion [173]. Additionally, IL-2 can enhance Fas-mediated apoptotic signaling, thereby promoting effector T cell death [174].

Temporal characteristics of functional switching: The functional switching of IL-2 exhibits distinct temporal characteristics. During the early stages of T cell response (early tumor development), IL-2 concentrations are relatively low and primarily exert proliferation-promoting antitumor effects; however, during the intermediate and late stages of tumor development, when cytokine accumulation and microenvironmental changes occur, the immunosuppressive and proapoptotic effects of IL-2 become dominant [175]. The molecular mechanisms underlying this switching involve differential expression of IL-2 receptors on different cell populations and the influence of other inhibitory factors in the TME.

#### IL-6

IL-6 is one of the most representative dual-function cytokines, the role of which in tumor development is highly dependent on tumor type, developmental stage, and microenvironmental status [176].

Protumor mechanisms: The protumor effects of IL-6 have been widely recognized and are primarily achieved through the following mechanisms: activation of the STAT3 signaling pathway, whereby IL-6 binding to its receptor activates the JAK-STAT3 pathway, promoting the expression of genes related to tumor cell proliferation, survival, and invasion [177]; induction of epithelial–mesenchymal transition (EMT), whereby IL-6 down-regulates E-cadherin expression while up-regulating vimentin and N-cadherin expression, thereby enabling tumor cells to acquire mesenchymal cell characteristics and enhance their invasive and metastatic capabilities [178]; maintenance of CSC properties, whereby IL-6 maintains the self-renewal capacity and pluripotency of CSCs through activation of the STAT3 and Notch signaling pathways [179]; recruitment of immunosuppressive cells, whereby IL-6 recruits immunosuppressive cells such as MDSCs and Tregs to infiltrate the TME, thus suppressing antitumor immune responses [180]; and promotion of angiogenesis, whereby IL-6 stimulates VEGF production, thereby facilitating tumor vascularization and providing nutritional support for tumor growth [181].

Antitumor mechanisms: However, under specific conditions, IL-6 can also exert antitumor effects: promotion of acute inflammatory responses, whereby in the early stages of tumor development, IL-6 activates acute inflammatory responses, promotes DC maturation and antigen presentation, and initiates antitumor immune responses [182]; enhancement of NK cell and CTL function, whereby appropriate levels of IL-6 activate NK cells and CTLs, thereby enhancing their cytotoxic capacity against tumor cells [183]; promotion of Th1-type immune responses, whereby under certain circumstances, IL-6 promotes Th1 cell differentiation and enhances cellular immune responses [184]; and regulation of B cell function, whereby IL-6 promotes B cell differentiation into plasma cells, thereby producing tumor-specific antibodies [185].

Regulatory factors for functional switching: The functional switching of IL-6 is primarily regulated by the following factors: concentration dependency, whereby low concentrations of IL-6 primarily exert antitumor effects, while high concentrations tend to promote tumor progression [186]; temporal characteristics, whereby IL-6 primarily exerts antitumor effects in the early tumor stages and transitions to protumor effects as tumors progress [187]; microenvironmental status, whereby IL-6 exerts antitumor effects under acute inflammatory conditions but promotes tumor progression under chronic inflammatory conditions [182]; and target cell type, whereby IL-6 primarily exerts immune activation effects on immune cells but mainly promotes proliferation and survival in tumor cells [185].

#### IL-7

IL-7 is a key cytokine essential for the development and maintenance of the adaptive immune system, playing crucial roles in the differentiation, proliferation, and survival of B cells and T cells; however, its role in tumor pathogenesis remains controversial [188].

Antitumor mechanisms: The antitumor effects of IL-7 are primarily achieved through the following pathways: First, by promoting the development and functional maintenance of antitumor immune cells, IL-7 can enhance and maintain the expression of the B lymphocyte-specific transcription factor EBF1, thereby promoting B cell production and antibody formation [189]; second, IL-7 regulates T cell survival, proliferation, differentiation, and activation, thus enhancing immune responses against pathogens and tumors by promoting naïve T cell differentiation into effector T cells and memory T cells [190]; third, IL-7 maintains NK cell homeostasis and activation status [191], while also promoting DC differentiation [192]; fourth, IL-7 exerts indirect antitumor effects by regulating the immune cell release of antitumor cytokines such as IFN-γ, IL-1β, IL-1α, and TNF-α [193–195]; finally, recent studies have found that IL-7 can also protect CD8^+^ T cells by blocking the immunosuppressive effects of the adenosine pathway, thus enhancing their cytotoxic activity against tumor cells [196].

Protumor mechanisms: The protumor effects of IL-7 are primarily manifested in directly promoting tumor cell proliferation and survival. IL-7 binds to the IL-7 receptor, activating multiple signaling pathways including JAK/STAT5, PI3K/AKT, and Ras/ERK, regulating the expression levels of Bcl-2 family genes, thereby promoting tumor cell proliferation and survival while inhibiting tumor cell apoptosis [197]. Additionally, IL-7 can rescue thymic CD4^+^CD8^+^ T cell subsets from apoptosis, which may potentially maintain tumor-associated immunosuppressive states under certain circumstances [198].

Tumor type-specific effects: The function of IL-7 is highly dependent on tumor type [199]. In hematological malignancies, such as acute lymphoblastic leukemia, IL-7 levels are typically markedly elevated, primarily exerting protumor effects [200]. In solid tumors, IL-7 primarily exerts immune-supportive effects, but its role may switch as tumor progression and immune exhaustion intensify [201].

#### IL-10

IL-10 was initially discovered as an immunosuppressive cytokine produced by Th2 cells that inhibits Th1 cell function; however, its role in tumor development is extremely complex, exhibiting pronounced duality [202].

Protumor mechanisms: The protumor effects of IL-10 have been widely acknowledged and are primarily mediated through the following mechanisms: Immunosuppressive effects: IL-10 induces potent immunosuppressive responses in macrophages and other APCs, primarily through transcriptional inhibition of cytokines, chemokines, MHC class II molecules, costimulatory molecules, and adhesion molecules [203–205]; and modulation of the TME and restriction of antigen presentation, thereby preventing effective T cell responses [206]. Recent studies using colorectal cancer liver metastasis organoid models have demonstrated that IL-10 promotes hepatic tumor metastasis by inducing programmed death-ligand 1 (PD-L1) expression and suppressing antitumor immune responses [207].

Antitumor mechanisms: However, an increasing body of research indicates that IL-10 also possesses antitumor properties: promoting the proliferation and activation of CD8^+^ T cells, enhancing their cytotoxicity and antitumor capacity [208]; alleviating the tumor-promoting effects mediated by cytokines such as IL-6 and IL-23 under chronic inflammatory conditions [209]; in the TME, engineered CAR-T cells expressing IL-10 can prevent T cell exhaustion by regulating the mitochondrial pyruvate carrier (MPC), thereby improving the mitochondrial health of CAR-T cells, promoting oxidative phosphorylation, and enhancing their proliferative capacity and effector functions [210]; and in multiple xenograft models, IL-10-expressing CAR-T cells can not only effectively eliminate orthotopic solid tumors but also induce the formation of stem cell-like memory T cells, thereby preventing tumor recurrence [211].

Cellular source dependency of functional switching: The functional switching of IL-10 largely depends on its cellular source [212]. IL-10 derived from Th2 cells and CD8^+^ T cells primarily exerts antitumor effects, while IL-10 derived from Tregs, MDSCs, and M2-type macrophages predominantly exhibits protumor functions [213]. This cellular source-dependent variability provides opportunities for precise modulation of IL-10 function [214].

#### IL-15

IL-15 was initially discovered as a T cell proliferation factor and plays a crucial role in the development and functional maintenance of the innate immune system, being particularly essential for the development, maturation, and activation of NK cells and NKT cells [215].

Antitumor mechanisms: The antitumor effects of IL-15 have been widely recognized, including stimulating T cell proliferation, generating CTLs, promoting B cell immunoglobulin synthesis, and facilitating NK cell generation and sustained survival [216]; and maintaining the survival and function of memory CD8^+^ T cells, thereby prolonging the duration of antitumor immune responses [217]. Recent studies have revealed that tumor cell-expressed IL-15 is crucial for the expansion, effector differentiation, and antitumor effects of innate lymphoid cell 1 (ILC1) and is capable of inducing natural antitumor immune responses in ILC1 [218].

Protumor mechanisms: However, an increasing number of studies indicate that IL-15 also possesses tumor growth-promoting activity under specific circumstances: In cutaneous T cell lymphoma (CTCL), increased methylation of the IL-15 promoter can drive tumor progression [219]; in human T lymphocytic leukemia, the viral Tax protein induces the production of IL-15 and IL-15Rα, establishing an IL-15 autocrine loop that leads to leukemia progression [220]; in multiple myeloma, malignant plasma cells exhibit high expression of functional IL-15 receptors and increased IL-15Rα chain expression, accompanied by autocrine IL-15 production; these characteristics promote the survival and proliferation of malignant plasma cells, enabling them to escape TME regulation [221].

Tumor type-specific expression: The function of IL-15 is highly dependent on tumor type and expression patterns. In solid tumors, IL-15 primarily exerts antitumor effects, and exogenous IL-15 therapy can markedly enhance the antitumor activity of NK cells and CD8^+^ T cells [222]. In hematological malignancies, particularly when IL-15 autocrine loops are present, IL-15 primarily promotes tumor cell survival and proliferation [223].

#### IL-17

The IL-17 family comprises 6 members from IL-17A to IL-17F, primarily produced by RORγt-expressing cells, including Th17 cells, γδT cell subsets, and innate lymphoid cells, as well as CD68^+^ macrophages, neutrophils, and mast cells [224–226].

Protumor mechanisms: The protumor effects of IL-17 are primarily manifested in the following: under chronic inflammatory conditions, IL-17 induces TME formation, promoting tumorigenesis and cancer progression through remodeling cellular matrix phenotypes, inducing inflammatory mesenchymal stem cell generation, and mobilizing bone marrow cells [227,228]; mediating CSC formation and EMT-like changes, IL-17 promotes EMT-like alterations in CSCs through the STAT3 pathway, serving as an important mechanism for tumor migration, invasion, and distant metastasis [229,230]; and establishing an immunosuppressive microenvironment, IL-17 can recruit immunosuppressive cells such as MDSCs and Tregs, suppressing antitumor immune responses [231].

Antitumor mechanisms: The antitumor effects of IL-17 are primarily achieved through the following pathways: recruiting and activating antitumor effector cells, IL-17 can recruit DCs, contributing to antitumor immunity in B16 melanoma [232] and Pten/Smad4-deficient lung cancer models [233]; stimulating antitumor cytokine production, enhancing immune responses by promoting the production of antitumor cytokines such as IFN-γ and TNF-α; and promoting the recruitment and activation of other effector immune cells, generating indirect antitumor activity [234,235].

Microenvironment-dependent functional switching: The switching between protumor and antitumor effects of IL-17 is closely related to the microenvironmental state. Under acute inflammatory conditions, IL-17 primarily promotes acute inflammatory responses and DC recruitment [236]. However, under chronic inflammatory conditions, sustained stimulation by IL-6, TGF-β, and IL-23 leads to persistently high levels of IL-17 secretion, at which point IL-17 primarily activates protumor gene programs, including the expression of angiogenic factors, matrix metalloproteinases, and EMT-related transcription factors [237].

#### IL-22

IL-22 similarly exhibits complex dual roles in tumor development, serving both as a barrier to tumorigenesis and progression, and as a promoter of tumor growth under certain conditions [238].

Antitumor mechanisms: Studies have revealed the presence of IL-22-producing innate lymphoid cells 3 (ILC3) within tertiary lymphoid structures formed in non-small cell lung cancer (NSCLC); these cells are more abundant in the early stages of tumor development, and tertiary lymphoid structures are considered to participate in the formation of antitumor immune responses [239]. IL-22 can also maintain epithelial barrier function, prevent the invasion of tumor-associated pathogens, and promote tissue repair and regeneration [240].

Protumor mechanisms: Two recent pivotal studies have revealed the prometastatic role of IL-22: First, IL-22-deficient mice and mice receiving IL-22 antibody treatment showed protection against liver and lung metastasis of colon cancer, while IL-22 overexpression promoted metastasis through a mechanism whereby IL-22 acts on endothelial cells, inducing endothelial aminopeptidase N expression and promoting endothelial permeability and cancer cell migration [241]; and second, IL-22 can induce high CD155 expression, excessively activating the CD226 receptor on NK cells, leading to reduced CD226 levels, impaired NK cell function, and consequently increased metastatic burden [242].

Development stage-related functional switching: IL-22 exhibits opposing functions at distinct phases of malignant progression. During initial periods of neoplastic transformation, particularly in tumors with abundant tertiary lymphoid structures, IL-22 primarily exerts antitumor effects [243]. However, as tumor progression and metastasis occur, the role of IL-22 shifts toward promoting tumor metastasis, particularly playing a crucial role in colorectal cancer liver metastasis [244].

#### IL-27

IL-27 is a cytokine with dual functions that has garnered marked attention in recent years, its functional complexity being particularly prominent across different tumor types and microenvironmental conditions.

Antitumor mechanisms: IL-27 is primarily secreted by activated macrophages and DCs, exerting multiple antitumor effects through activation of STAT1 and STAT3 signaling pathways: it directly inhibits tumor cell proliferation; induces cell cycle arrest and apoptosis; suppresses VEGF production, thereby blocking tumor angiogenesis; and enhances the cytotoxic functions of NK cells and CTLs [245].

Protumor mechanisms: However, recent studies have revealed that IL-27 exhibits protumor activity in certain tumor types [246]. Studies have demonstrated that under specific microenvironmental conditions, IL-27 can promote tumor cell survival and proliferation, induce the expression of immunosuppressive molecules, and inhibit antitumor T cell functions [246]. This functional switch may be closely associated with factors such as tumor type, microenvironmental oxygen concentration, and the presence of other coexisting cytokines.

Microenvironment-dependent functional differences: The functional switch of IL-27 is highly dependent upon the state of the TME. In Th1-type microenvironments rich in IFN-γ and IL-12, IL-27 primarily exerts antitumor effects [247]; conversely, in immunosuppressive microenvironments rich in IL-10 and TGF-β, IL-27 may switch to exert protumor effects [248].

### IFN family

IFN-γ consistently plays a dual role in regulating both protumor and antitumor immune functions within the TME, with its functional manifestation being highly dependent on concentration, microenvironmental conditions, and target cell types [249].

Antitumor mechanisms: The antitumor effects of IFN-γ represent its most classical function: as a cytotoxic cytokine, IFN-γ acts with granzyme B and perforin to directly induce tumor cell apoptosis; it activates the JAK-STAT1-caspase signaling pathway, with high-dose IFN-γ capable of inducing apoptosis in NSCLC cell lines [250]; it interacts with stromal fibroblasts to down-regulate VEGF-A expression, inducing vascular destruction and promoting tumor tissue necrosis [251,252]; and it enhances MHC class I molecule expression, improving tumor cell immunogenicity and promoting tumor antigen presentation [253].

Protumor mechanisms: However, IFN-γ also exhibits protumor effects under specific conditions: it induces the synthesis of immune checkpoint molecules and indoleamine 2,3-dioxygenase, thereby activating multiple immunosuppressive mechanisms [254]; low concentrations of IFN-γ can enhance the survival rate of circulating tumor cells and augment their metastatic potential; and it induces lymphatic endothelial cells to express PD-L1, restricting the migration of CTLs from the peritumoral space to the TME and suppressing antitumor immune function [255].

Concentration-dependent functional switching: The functional switching of IFN-γ is closely related to its concentration within the TME. The accumulation of antigen-specific T cells increases the concentration of IFN-γ in tumor tissues, and when the concentration reaches a certain threshold, its immunosuppressive effects begin to predominate. This concentration-dependent effect explains why immune checkpoint inhibitors may have limited efficacy in some tumors with abundant T cell infiltration.

### TGF family

TGF-β is one of the most archetypal bifunctional cytokines, exhibiting diametrically opposite functions at different stages of tumor development, thus reflecting the high plasticity of cytokine functions.

Antitumor mechanisms: In normal tissues and early-stage tumors, TGF-β primarily exerts tumor suppressive effects: By inhibiting cell proliferation, TGF-β activates the Smad2/3 signaling pathway to induce the expression of cell cycle inhibitors such as p21, thereby arresting cell cycle progression [256]; promoting apoptosis by activating the expression of proapoptotic genes; suppressing growth factor production, thereby reducing tumor-associated pro-proliferative signals; and maintaining immune homeostasis to prevent excessive inflammatory responses from inducing tumor formation [257].

Protumor mechanisms: During tumor progression, the TGF-β signaling pathway becomes dysregulated or undergoes mutations, subsequently promoting tumor development: facilitating extracellular matrix remodeling to create favorable conditions for tumor invasion and metastasis [258]; stimulating angiogenesis to provide nutritional support for tumor growth; and suppressing antitumor T cell immune functions, since TGF-β is a key factor in Treg development and functional maintenance, thus creating a highly immunosuppressive tumor environment [259,260]. Recent studies have revealed that TGF-β markedly inhibits mitochondrial electron transport chain complex V activity through a Smad2-dependent mechanism, thereby impairing the ability of CD4^+^ T cells to produce IFN-γ [261].

Molecular mechanisms of functional switching: The key to TGF-β functional switching lies in alterations of its signaling pathways. In normal cells, TGF-β signaling can normally activate tumor suppressor genes; however, in tumor cells, due to Smad4 loss, Smad2/3 mutations, or other signaling pathway abnormalities, TGF-β loses its antiproliferative capacity and instead activates the expression of protumor genes. This switching involves complex epigenetic regulation, including methylation of promoter regions, histone modifications, and regulation by noncoding RNAs [262].

### CSF family

GM-CSF is a critical cytokine that regulates the proliferation and differentiation of bone marrow stem cells and mature granulocytes, demonstrating distinct dual roles in tumor immunity [263].

Antitumor mechanisms: The antitumor effects of GM-CSF are primarily manifested through several mechanisms: first, promoting hematopoietic function, where recombinant human GM-CSF can be used to prevent and treat leukopenia, bone marrow hematopoietic dysfunction, and other diseases [264]; second, serving as an immune enhancer, GM-CSF can promote DC differentiation, maturation, and antigen presentation function, enhancing antitumor immune responses [265,266]; and third, activating the antitumor functions of macrophages and neutrophils, thereby enhancing their phagocytic and cytotoxic capabilities.

Protumor mechanisms: However, recent studies have revealed that GM-CSF can also promote tumor progression under certain circumstances [263]. Specifically, in patients with extranodal natural killer/T cell lymphoma, GM-CSF treatment led to rapid disease progression, with mechanisms involving STAT5 mutations and JAK2 hyperphosphorylation, which up-regulated PD-L1 expression in tumor cells and impaired tumor immune surveillance function [266]. Similarly, in gastric cancer, tumor-derived GM-CSF activates neutrophils through the JAK2/STAT3 signaling pathway, inducing the generation of PD-L1^+^ neutrophils, which suppress T cell immune function and promote tumor growth and progression [267].

Impact of genetic mutations on function: The functional switch of GM-CSF is closely associated with specific genetic mutations. STAT5 mutations and JAK2 hyperactivation represent key molecular events that enable GM-CSF to exert protumor effects, as these mutations alter GM-CSF downstream signaling, thereby leading to aberrant expression of immunosuppressive molecules such as PD-L1.

### TNF family

TNF-α, which was named for its ability to induce necrosis of transplanted sarcomas at high concentrations, is one of the most extensively studied bifunctional cytokines [268,269].

Antitumor mechanisms: The antitumor effects of TNF-α are achieved through multiple pathways: (a) vascular destructive effects, in which local application of exogenous TNF-α can promote tumor vascular destruction, leading to indirect tumor cell necrosis [270]; (b) increased vascular permeability, which facilitates chemotherapeutic drug accumulation at tumor sites and produces synergistic effects with liposome-mediated chemotherapy [271]; (c) direct cytotoxicity, whereby high concentrations of exogenous TNF-α can directly induce tumor cell apoptosis through activation of death receptor signaling pathways [268]; and (d) immune activation effects, whereby TNF-α can activate immune cells such as macrophages and DCs, enhancing antitumor immune responses.

Protumor mechanisms: The protumor effects of TNF-α are primarily associated with chronic inflammation: (a) sustained TNF-α signaling activates the NF-κB pathway, promoting proinflammatory gene expression and establishing an inflammatory microenvironment favorable for tumor growth; (b) stimulation of angiogenesis and matrix remodeling, thereby creating conditions for tumor invasion and metastasis; and (c) recent studies have demonstrated that in melanoma patients receiving adoptive T cell immunotherapy, TNF-α can induce tumor cell dedifferentiation, leading to decreased expression of melanoma markers, reduced immunogenicity, and promotion of tumor recurrence [272].

Receptor-mediated differential regulation: The dual functions of TNF-α are partially attributed to the different roles of its 2 receptors, TNFR1 and TNFR2. TNFR1 is widely expressed and primarily mediates proinflammatory and proapoptotic signals, whereas TNFR2 exhibits restricted expression, is overexpressed in tumor cells, and mainly promotes tumor cell proliferation and survival [273–275].

Treatment-related functional complexity: During immunotherapy, the role of TNF-α is particularly complicated. Moderate elevation of TNF-α helps activate antitumor immunity, but excessive or sustained TNF-α production may lead to tumor cell dedifferentiation and immune escape. This duality poses marked challenges for the application of TNF-α in cancer therapy [276].

The existence of bifunctional cytokines reveals the complexity and dynamics of tumor immune regulation. The functional switching of these cytokines is regulated by multiple factors, including concentration-dependent effects, temporal characteristics, microenvironmental status, target cell types, and signaling pathway states. Understanding these regulatory mechanisms is of great importance for developing precise cytokine-targeted therapeutic strategies.

In clinical applications, it is essential to comprehensively consider factors such as tumor type, developmental stage, and microenvironmental characteristics to precisely regulate the actions of these bifunctional cytokines, thereby maximizing therapeutic efficacy while minimizing adverse reactions. This functional duality represents both challenges and opportunities. Through an in-depth understanding of the mechanisms of action and regulatory patterns of bifunctional cytokines, we may develop more precise and effective tumor immunotherapy strategies, ultimately bringing better therapeutic outcomes for cancer patients (Table 1).

## Challenges in Cytokine-Based Cancer Immunotherapy

**Fig. 4.**
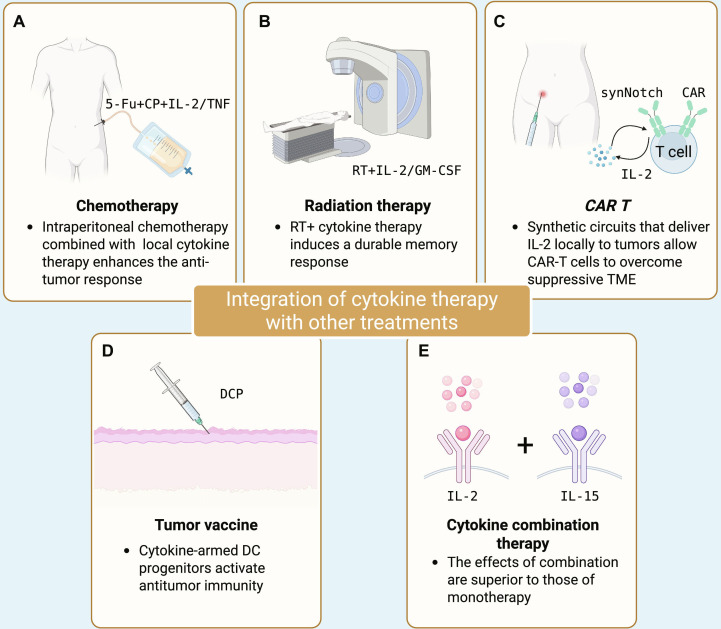
Personalized treatment strategies combining cytokines with other therapies. (A) Intraperitoneal chemotherapy: Combining intraperitoneal chemotherapy with local cytokine therapy enhances the antitumor response. This localized approach maximizes the efficacy of chemotherapy by leveraging the immune-stimulating effects of cytokines. (B) RT + cytokine therapy: The combination of radiation therapy with cytokine therapy induces a durable memory response. This approach aims to not only reduce tumor size but also stimulate long-term immune memory against the tumor. (C) Synthetic circuits: Using synthetic circuits that deliver IL-2 locally to tumors allows CAR-T cells to overcome the suppressive tumor microenvironment (TME). This method enhances the efficacy of CAR-T cell therapy by providing necessary cytokines directly at the tumor site. (D) Cytokine-armed DC progenitors: Tumor vaccines utilizing cytokine-armed dendritic cell progenitors activate antitumor immunity. This approach aims to prime the immune system to recognize and attack tumor cells more effectively. (E) Combination of cytokines: The effects of combining different cytokines are superior to those of monotherapy. This strategy takes advantage of the synergistic effects of multiple cytokines to enhance the overall antitumor response. These personalized treatment strategies integrate cytokine therapy with other modalities to maximize therapeutic efficacy and tailor treatments to individual patient needs. Abbreviations: 5-Fu, 5-fluorouracil; CAR-T, chimeric antigen receptor T cells; CP, cisplatin; DC, dendritic cells; DCP, dendritic cell progenitors; GM-CSF, granulocyte-macrophage colony-stimulating factor; IL-2, interleukin-2; IL-15, interleukin-15; RT, radiation therapy; synNotch, synthetic notch; TME, tumor microenvironment; TNF, tumor necrosis factor.

Given that cytokines play crucial regulatory roles in both tumorigenesis and tumor cell elimination, the clinical use of recombinant cytokines for cancer treatment has an extensive history. Currently, cytokine-based immunotherapy can be considered the first successful cancer immunotherapy approach, capable of inducing durable antitumor immunity in humans [277]. In 1986, recombinant IFNα-2b received regulatory authorization for managing hairy cell leukemia [278]. In 1992, the Food and Drug Administration (FDA) granted therapeutic approval for high-dose IL-2 in treating advanced renal cell carcinoma, and in 1998, it broadened this indication to encompass metastatic melanoma [279] (Fig. 4 and Table 2).

**Table 2.** Principal cytokine or cytokine receptor antagonists investigated in clinical settings as potential cancer therapies

Target	Purpose	Status	Phase	NCT no.	Other combination therapies
TNF	To identify the best TNFi to be combined with ICI in advanced melanoma patients	Recruiting	I	NCT05867004	ICIs
TNFR	To test a mAb of TNFR2 alone and with ICI in advanced solid tumors	Recruiting	I/II	NCT06205706	
IL-6	To test the addition of an anti-IL6 to standard induction chemotherapy for high-risk AML	Recruiting	I	NCT05697510	Chemotherapies and CAR T
IL-6R	To confirm the IL-6R antagonist will be safe and effective at inducing tumor response in children with residual adamantinomatous craniopharyngioma	Recruiting	II	NCT05233397	
IL-1β	To evaluate the IL-1β antagonist alone and in combination with ICI in adult patients with advanced solid malignancies	Recruiting	I	NCT05441046	Chemotherapies and ICIs
IL-1R	To test a mAb of IL-1R alone and with chemotherapy in triple-negative breast cancer	Recruiting	I/II	NCT05181462	
TGF-β	To assess the safety, tolerability, pharmacokinetics, and efficacy of a TGF-β antagonist in patients with advanced solid tumors	Recruiting	I/II	NCT06223308	Targeted agents and ICIs
TGF-βR1	To further evaluate the safety, tolerability, and preliminary antitumor activity of a TGF-βR1 antagonist	Active, not recruiting	I	NCT05228600	
CCR2	To evaluate the safety and tolerability of a CCR2 antagonist in combination with chemotherapy	Completed	I	NCT02732938	Targeted agents and ICIs
CCR4	To confirm mogamulizumab together with extracorporeal photopheresis may work better in treating patients with Sezary syndrome or mycosis fungoides compared to either therapy alone	Recruiting	I/II	NCT04676087	Targeted agents and ICIs
CCR5	To test the efficacy of a mAb to CCR5 in patients with CCR5^+^ locally advanced or metastatic solid tumors	Unknown	II	NCT04504942	ICIs
CXCR4	To confirm ICI and CXCR4 antagonist may work better in treating patients with pancreatic cancer	Completed	II	NCT02907099	Targeted agents and ICIs
CSF1R	To evaluate the tolerability, pharmacokinetics, pharmacodynamics and preliminary efficacy of a mAb of CSF1R in patients with advanced solid tumors	Recruiting	I	NCT05212896	

TNF, tumor necrosis factor; TNFi, TNF inhibitor; ICI, immune checkpoint inhibitor; TNFR, TNF receptor; mAb, monoclonal antibody; IL-6, interleukin-6; IL-6R, IL-6 receptor; AML, acute myeloid leukemia; CAR T, chimeric antigen receptor T cell; IL-1β, interleukin-1 beta; IL-1R, IL-1 receptor; TGF-β, transforming growth factor beta; TGFβR1, TGF-beta receptor 1; CCR2, C-C motif chemokine receptor 2; CCR4, C-C motif chemokine receptor 4; CCR5, C-C motif chemokine receptor 5; CXCR4, C-X-C motif chemokine receptor 4; CSF1R, CSF1 receptor; NCT, National Clinical Trial

However, cytokines as therapeutic agents face numerous limitations in clinical applications, including cytokine pleiotropy, complex biological properties, poor drug-like characteristics, and severe dose-limiting toxicities [277]. In fact, the roles that cytokines play in tumorigenesis depend specifically on tumor type and the TME, suggesting that researchers may need to employ cytokine activators or antagonists that target specific TMEs. Particularly noteworthy are cytokines with dual functions, which may exert different functions at different stages of tumor progression. For example, the aforementioned IL-2 signaling exhibits distinctly different roles at different stages of tumor progression: it exerts antitumor effects in early-stage tumors, while primarily exerting immunosuppressive effects in advanced-stage tumors; it is currently established that IFN-γ, TNF-α, and the IL-1 family similarly play dual roles in the cancer immunity cycle [26].

### Therapeutic challenges of spatial distribution heterogeneity of cytokines

Another marked challenge confronting cytokine therapy is the heterogeneity of their distribution across different anatomical sites. This complexity in spatial distribution presents considerable difficulties in developing effective targeted therapeutic strategies [280].

Current clinical practice primarily relies on serum cytokine levels as biomarkers to guide therapeutic decisions; however, this approach has obvious limitations. Serum levels may not accurately reflect local cytokine activity within tumor tissues, particularly within the TME, where local concentrations and activities of cytokines may differ markedly from circulating levels [281]. For patients with multiple metastases, cytokine expression patterns at different metastatic sites may be completely different [282]; moreover, there is currently a lack of effective methods to comprehensively assess this spatial heterogeneity.

The spatial distribution heterogeneity of cytokines renders treatment selection even more complex. If a particular cytokine exerts antitumor effects in the primary tumor but primarily exhibits protumor effects at metastatic sites, systemic cytokine-modulating therapy may yield contradictory effects. In such scenarios, treatment regimens based on single-site biopsy results may not adequately address the patient’s overall condition [283].

In-depth investigation of cytokine spatial distribution encounters both technical and ethical challenges. Multisite biopsies not only are technically complex but also elevate patient risk and discomfort [284]. There is currently a lack of standardized multisite assessment methods, which not only affects the comparability of clinical studies but also constrains the development of individualized treatment regimens [285]. While liquid biopsy technology offers the possibility of noninvasive detection, its accuracy in reflecting local tissue cytokine status still requires further validation.

### Pharmacological challenges of cytokine therapy

Although cytokines and cytokine receptors have been extensively studied as therapeutic targets for cancer treatment over the past 40 years, the clinical translation rate remains relatively low [10]. On one hand, cytokine monotherapy has disadvantages including short half-life in circulation, poor biodistribution, redundant target pathways, and excessive toxicity; on the other hand, these limitations are also associated with the immunosuppressive effects, environmental dependency, and pleiotropic effects of cytokines [286].

Cytokines are a class of small molecular proteins naturally produced by the body that play an indispensable role in regulating the activation, proliferation, and functional performance of immune cells. Given their potent immunostimulatory effects, cytokines are recognized as ideal candidate drugs for enhancing overall immune function and improving antitumor immune responses [287]. However, while exerting antitumor effects, cytokines are often accompanied by dose-limiting toxic reactions, such as cytokine release syndrome (CRS) and vascular leak syndrome, which greatly restrict the clinical application and widespread adoption of cytokine-based drugs [288].

Taking IL-2 as an example, it is currently the only cytokine drug approved by the FDA for advanced renal cell carcinoma and melanoma [279]. However, high-dose IL-2 treatment is often accompanied by severe adverse effects, such as hypotension, pulmonary edema, and cardiac arrhythmias, requiring close monitoring and management in an intensive care unit (ICU) setting, which not only increases medical costs but also compromises patients’ quality of life [289]. Similarly, although IL-15 demonstrates superior antitumor efficacy and safety compared to IL-2 in vivo, varying degrees of adverse effects are still observed in clinical trials [290]. Therefore, how to reduce adverse effects while preserving the antitumor activity of cytokines has become a key focus and challenge in current cytokine drug development.

### Limitations of anticytokine antibody therapy

On the other hand, the primary dilemmas of anticytokine monoclonal antibody (mAb) therapy lie in its high development and production costs, potential immunogenic reactions, substantial individual variations in therapeutic efficacy, risks of severe adverse effects, and possible immune function suppression in certain circumstances, all of which collectively limit its widespread application and long-term therapeutic efficacy. Nevertheless, anticytokine mAbs still represent an emerging strategy in the field of tumor immunotherapy. Although most clinical trials of such drugs are currently in early stages and primarily involve dose exploration and safety assessment in advanced tumor patients, research in this direction still holds tremendous potential [291].

Results from early clinical trials demonstrate that the efficacy of anticytokine mAb monotherapy is relatively limited, with some drugs even failing to demonstrate clear antitumor activity. This may be attributed to the following factors: advanced tumor patients typically have high tumor burden, and their immune system function is often markedly suppressed, making it difficult for single cytokine antagonism to completely reverse the tumor-associated immunosuppressive microenvironment; complex cytokine networks exist in the TME, with functional redundancy and compensatory actions among different cytokines; and some tumors can escape the therapeutic effects of anticytokine mAbs by up-regulating other immunosuppressive molecules (such as PD-L1) [292].

Despite the limited efficacy of cytokine antagonist monotherapy, many early clinical trials have confirmed the good safety and tolerability of anticytokine mAbs. Compared to the common toxic reactions associated with cytokine-type drugs (such as CRS), anticytokine mAb therapy exhibits a lower incidence of adverse events, which are mostly mild symptoms, with rare occurrences of dose-limiting toxicity or severe adverse reactions [293].

### Development of combination therapeutic strategies

Given the limitations of monotherapy and their favorable safety profiles, anticytokine mAbs are currently primarily utilized in combination with other antitumor therapeutic regimens to achieve synergistic and additive effects. For instance, combining anti-IL-6 and anti-IL-8 mAbs with immune checkpoint inhibitors can simultaneously alleviate tumor-associated inflammation and immunosuppression [294], and combining anti-IL-4 and anti-IL-13 mAbs with tumor vaccines can reduce vaccine-induced Th2-type responses and promote Th1-type antitumor immunity [295]. Furthermore, anticytokine mAbs may additionally be integrated with standard treatment modalities including chemotherapy, radiotherapy, and targeted therapy to amplify treatment outcomes while minimizing adverse reactions [296].

Therefore, the effective development of cytokine-based combination immunotherapies is of paramount importance. Cytokine therapy can unleash the potential of other therapeutic modalities, thereby enabling drug-resistant patients to benefit from treatment. Research by Wang and colleagues reported an immune-priming therapy consisting of a single-dose combination treatment composed of pan-tumor targeting antibody surrogates, half-life-extended IL-2, and PD-1 antibodies. This strategy can recruit NK cells and macrophages to infiltrate tumor sites, promote lymphocyte homing and activation, and induce tumor vascular normalization, thereby establishing a foundation for subsequent immune checkpoint blockade (ICB) therapy and enabling ICB to effectively eliminate established large-volume tumors [297]. Studies have also demonstrated that local induction of cytokine (such as IL-2) production can effectively overcome immunosuppression, enhance CAR-T cell infiltration, and efficiently eliminate various immunologically excluded tumor models (pancreatic cancer and melanoma) [298].

To overcome the toxic side effects of systemic IL-2 administration, researchers have developed various local delivery strategies in recent years. These approaches aim to achieve high-concentration accumulation of IL-2 at tumor sites while minimizing systemic toxicity. Local delivery strategies for IL-2 include direct intratumoral injection, drug-loaded nanoparticle targeted delivery, and engineered long-acting IL-2 formulations. Studies have shown that these local delivery methods not only markedly enhance antitumor immune responses but also effectively reduce systemic side effects, providing new insights and directions for the application of IL-2 in cancer immunotherapy [299]. The combined application of these local delivery strategies with CAR-T cell therapy represents an important developmental direction in the field of cytokine therapy (Fig. 4).

## Summary and Future Perspectives

Despite marked advances in understanding cytokine functions in the TME, several critical challenges remain. The extensive tumor heterogeneity and the multifaceted nature of cytokine functions have resulted in fragmented knowledge of cytokine regulatory networks. Different cytokines exhibit varied functional manifestations across tumor types, while the same cytokine may exert opposing effects at different stages of tumor development, thereby highlighting the complexity of their regulatory mechanisms [300,301].

Future investigations should prioritize comprehensive studies of cytokine networks through multiomics approaches, including single-cell RNA sequencing, proteomics, and epigenomics [302,303]. This integrative approach will facilitate the elucidation of cytokine expression profiles across different tumor types and developmental stages, thereby establishing their associations with malignant phenotypes and immune microenvironment characteristics [25]. Dynamic immune monitoring systems should be developed to track real-time changes in cytokine networks during treatment, thus enabling personalized therapy optimization [304].

Research efforts should focus on elucidating the signal transduction pathways, downstream effector molecules, and cross-regulatory mechanisms of different cytokines in specific tumor models [291]. Key genetic and epigenetic factors influencing cytokine function require investigation to decipher the regulatory mechanisms underlying functional heterogeneity [32]. Additionally, understanding how various therapeutic modalities (radiotherapy, chemotherapy, targeted therapy, and immunotherapy) modulate cytokine function is crucial for optimizing therapeutic combination strategies.

The short half-life and the potential for systemic toxicity of cytokines necessitate the development of novel targeted delivery approaches. Promising strategies include nanomaterial-based carriers for controlled release, cell engineering techniques for continuous in vivo cytokine production, and cytokine fusion proteins conjugated with specific receptors or antibodies for precise delivery to targeted cell populations [22].

Given the substantial individual differences in cytokine networks among patients, personalized approaches are essential. This requires a comprehensive assessment of baseline cytokine expression profiles and TME characteristics to identify appropriate therapeutic targets. Treatment regimens should be dynamically adjusted based on patient responses, with potential integration with other immunotherapeutic agents or conventional therapies to achieve synergistic effects [26].

Cytokines represent indispensable regulatory factors in the TME with marked implications for both tumor development and cancer immunotherapy. While considerable scientific and clinical challenges remain due to their complex and plastic mechanisms of action, ongoing advances in basic research and technological innovations promise increasingly favorable prospects for their clinical applications. The development of systematic research approaches, mechanistic insights, targeted delivery strategies, and personalized treatment regimens will be crucial for realizing the full therapeutic potential of cytokine-based cancer immunotherapy.
